# Nucleic Acid and Non-Nucleic Acid-Based Reprogramming of Adult Limbal Progenitors to Pluripotency

**DOI:** 10.1371/journal.pone.0046734

**Published:** 2012-10-08

**Authors:** Sowmya Parameswaran, Sudha Balasubramanian, Norbert Babai, Carolina B. DelDebbio, Donald W. Harms, Channabasavaiah B. Gurumurthy, Mahendra S. Rao, John G. Sharp, Iqbal Ahmad

**Affiliations:** 1 Department of Ophthalmology and Visual Sciences, University of Nebraska Medical Center, Omaha, Nebraska, United States of America; 2 Department of Genetics Cell Biology and Anatomy, University of Nebraska Medical Center, Omaha, Nebraska, United States of America; 3 Center for Regenerative Medicine,National Institutes of Health, Bethesda, Maryland, United States of America; National Eye Institute, United States of America

## Abstract

Reprogramming somatic cells to a pluripotent state by nucleic acid based (NAB) approaches, involving the ectopic expression of transcription factors, has emerged as a standard method. We recently demonstrated that limbal progenitors that regenerate cornea are reprogrammable to pluripotency by a non-NAB approach through simple manipulation of microenvironment thus extending the possible therapeutic use of these readily accessible cells beyond the proven treatment of corneal diseases and injury. Therefore, to determine the validity and robustness of non-cell autonomous reprogramming of limbal progenitors for a wider clinical use, here, we have compared their reprogramming by non-NAB and NAB approaches. We observed that both approaches led to (1) the emergence of colonies displaying pluripotency markers, accompanied by a temporal reciprocal changes in limbal-specific and pluripotency gene expression, and (2) epigenetic alterations of Oct4 and Nanog, associated with the de-novo activation of their expression. While the efficiency of reprogramming and passaging of re-programmed cells were significantly better with the NAB approach, the non-NAB approach, in contrast, led to a regulated reprogramming of gene expression, and a significant decrease in the expression of *Hormad1*, a gene associated with immunogenic responses. The reprogramming efficiency by non-NAB approach was influenced by exosomes present in conditioned medium. Cells reprogrammed by both approaches were capable of differentiating along the three germ lineages and generating chimeras. The analysis suggests that both approaches are effective in reprogramming limbal progenitors but the non-NAB approach may be more suitable for potential clinical applications by averting the risk of insertional mutagenesis and immune responses associated with the NAB approach.

## Introduction

**Figure 1:**
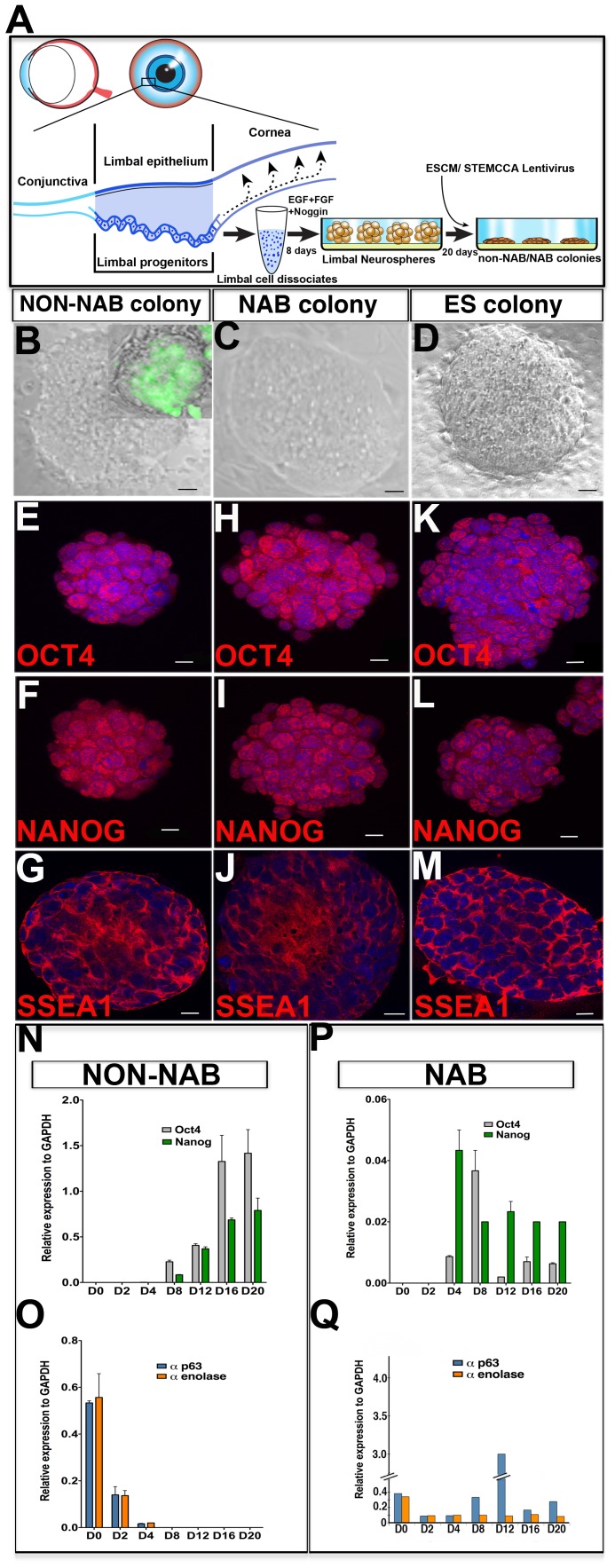
Induction of iPS cell phenotypes in limbal progenitors by the non-NAB and NAB methods. A schematic of the method (A) shows the location of progenitors in the basal layer of the circular limbal epithelium surrounding the cornea. Progenitors in limbal cell dissociated give rise to neurospheres in the presence of EGF+FGF2+Noggin, which when cultured either in the presence of embryonic stem cell conditioned medium (ESCM) or transduced with STEMCCA lentivirus generate non-NAB or NAB iPS colonies between 15–20 days. Limbal iPS colonies generated by the non-NAB method (B) or through NAB method (C) generate colonies, morphologically similar to mouse ES cell colonies (D). GFP-expressing mouse limbal progenitors subjected to non-NAB reprogramming resulted in GFP-positive colonies confirming the source of colonies to be mouse limbal cells and not contaminant ES cells (Figure 1B (inset)). Cells in the colonies obtained by either non-NAB (E-G) or NAB (H-J) method expressed immunoreactivities corresponding to pluripotency markers OCT4, NANOG, and SSEA1, similar to those in ES cell colonies (K-M). Expression analysis by Q-PCR revealed a temporal induction of pluripotency genes (*Oct 4* and *Nanog*) (N) and attenuation of limbal progenitor-specific genes (*α-p63* and *α-enolase*) (O) during the generation of colonies by non-NAB method. Induction of *Oct4* and Nanog (P) and attenuation of *α-p63* and *α-enolase* (Q) were also observed in colonies generated by the NAB method but appeared less regulated, compared to the non-NAB method. Scale bar: B- D 50 µm;E–M 20 µm.

Direct reprogramming of somatic cells to induced pluripotent stem cells (iPS cells) by forced expression of defined transcription factors (TFs) is a significant breakthrough in the generation of patient specific cells to understand disease processes, and ultimately for treating them by autologous cell therapy. However, the initial methods employing viral vectors for over expressing TFs has represented a barrier to therapeutic applications of iPS cells owing to the risk of insertional mutagenesis [1] and immunogenic responses [2]. The nucleic acid-based (NAB) approaches including the use of non-integrating viral vectors [3], transient transfection of plasmids [4], synthetic mRNAs [5], and miRNAs [6] and non-nucleic acid based (non-NAB) approaches including the transduction of recombinant proteins [7], [8], and application of ES cell extracts [9] have emerged as alternative methods of reprogramming. However, the possibility of a facile non-NAB method of reprogramming emerged based on the observations that the number of transcription factors for reprogramming could be progressively decreased depending upon cell sources and culture conditions. For example, while reprogramming of adult somatic cells generally require four TFs, stem cells and progenitors can be reprogrammed by ectopic expression of only one TF, Oct4 [10] and the efficiency of re-programming can be increased by small molecules [11], [12]. In support of the premise, we demonstrated that somatic progenitors can be reprogrammed to pluripotency by a non-NAB approach that involved influencing the genome of the target cells non cell-autonomously by simple alteration of the microenvironment [13]. This approach has a precedence in the maintenance of pluripotency of mouse embryonic stem (ES) cells *in vitro* in the presence of embryonal carcinoma (EC) cell conditioned medium [14], loss of pluripotency and differentiation along neuronal lineage of ES cells in low density culture [15], and more recently observed metastable states of inner cell mass (ICM), ES and epiblast stem (EpiS) cells that allow reversions under epigenetic influence [16]. The target cells for reprogramming were progenitors that regenerate cornea, located in the basal layer of the circumscribing limbal epithelium (Figure. 1A). These cells have been successfully used in autologous stem cell therapy to treat blindness due to corneal injury and diseases [17]. Besides their easy accessibility, they readily de-differentiate into neural progenitors [18] when removed from their niche in the presence of Noggin, and endogenously express three of the four pluripotency factor genes, *Klf4*, *Sox2* and *c-Myc* [13]. The inducers were mouse ES cells. Here, we have compared reprogramming by the NAB and non-NAB approaches to validate and determine the robustness of non-cell autonomous approach to induce pluripotency in limbal progenitors. Both approaches caused the limbal progenitors to generate colonies, expressing pluripotency markers, with temporal decrease and increase in limbal-specific and pluripotency genes, respectively, and epigenetic alterations of *Oct4* and *Nanog* genes, associated with the de-novo reprogramming of their expression. The efficiency of reprogramming and passaging of re-programmed cells were better with the NAB approach, but the non-NAB approach, in contrast, led to a regulated reciprocal alteration in the expression of limbal specific and pluripotency genes, and a significant decrease in the expression of *Hormad1*, a gene associated with immunogenic responses. The efficiency of reprogramming by non-NAB approach was influenced by exosomes present in ES cell conditioned medium. Cells reprogrammed by both approaches were capable of differentiating along the three germ lineages and generating chimeras. The analysis suggests that the non-NAB approach may be more suitable for potential clinical applications, given it does not suffer from the risk of causing insertional mutagenesis and may not elicit immunogenic responses as does the NAB approach.

## Materials and Methods

The study was approved by the Institutional Animal Care and Use Committee (IACUC), at University of Nebraska Medical Center (protocols #97-100-08FC and #95-005-09FC), and animals were housed and bred in the Department of Comparative Medicine at University of Nebraska Medical Center.

### Neurosphere Generation

Dissection and enrichment of limbal epithelium progenitors was performed as previously described [18]. Briefly, eyes of adult mouse strains C57BL/6J, 129 SvJ were enucleated in Hank’s balanced salt solution. The limbal region was dissected and serially incubated in 0.05% trypsin (Sigma) for 45 minutes, in 78 U/ml of collagenase (Sigma) for 27 minutes, and finally in 38 U/ml of hyaluronidase (Sigma) for 30 minutes, all at 37°C, followed by trituration. Dissociated cells were cultured in DMEM: F12 (Gibco) supplemented with 1X N2 supplement, 20 ng/ml of EGF (R & D systems), 10 ng/ml of bFGF(R & D systems) and 100 ng/ml of Noggin (R & D systems), at a density of 10^5^ cells/cm^2^. After 4 days, resulting neurospheres were trypsinized and plated to generate secondary neurospheres. At the end of the 8^th^ day the secondary neurospheres were subjected to iPS cell induction.

**Figure 2:**
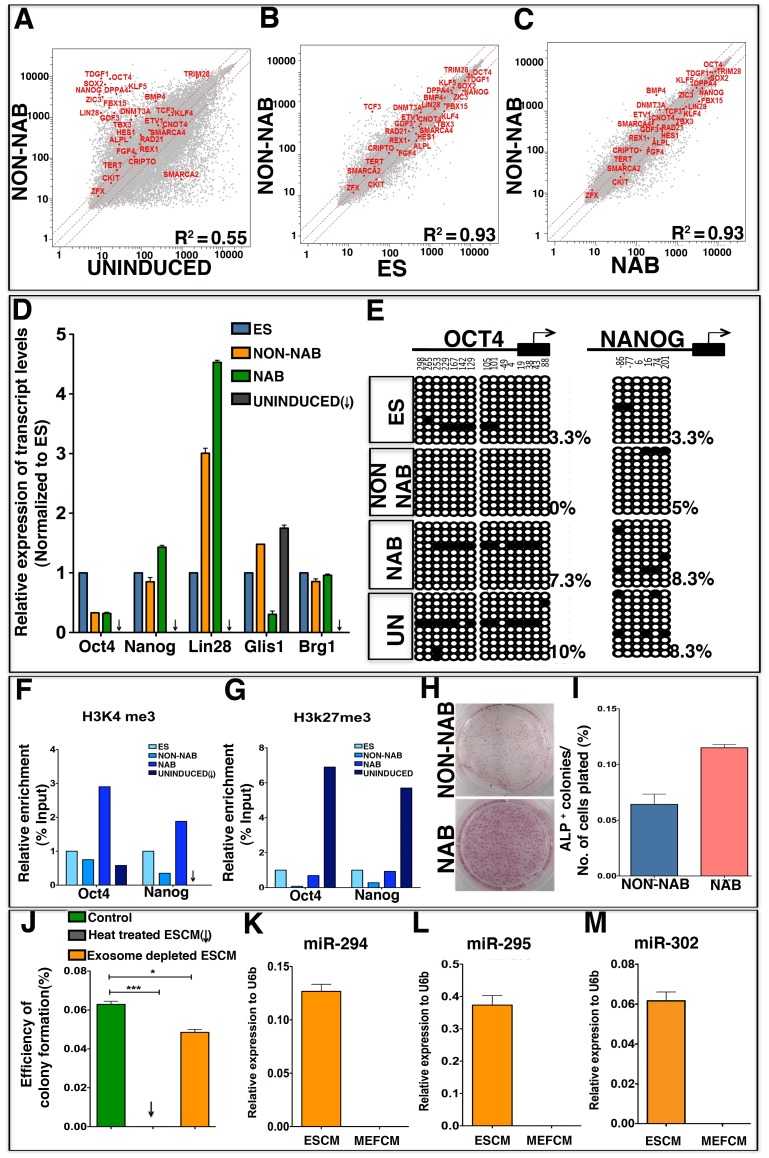
Reprogramming of limbal progenitors by the non-NAB method. Microarray analyses of global gene expression revealed induction of key pluripotency network genes in colonies generated by the non-NAB method, compared to un-induced cells (A), ES colonies (B) and NAB colonies (C). Red line indicates linear equivalent and two fold change in expression levels between samples. Q-PCR analyses revealed the expression of key pluripotency and other related genes, *Oct4*, *Nanog*, *Lin28*, *Glis1* and *Brg1* in both non-NAB and NAB colonies as in ES colonies, albeit at different levels suggesting that programming is similar but not identical (D). Bisulfite sequencing, carried out on genomic DNA derived from colonies obtained by different methods revealed a decrease in the number of methylated CpG dinucleotides in the *Oct4* and *Nanog* promoters in non-NAB and NAB colonies, compared to those in the un-induced limbal progenitors (E). Analysis of histone methylation status of *Oct4* and *Nanog* promoters revealed enrichment of H3K4me3 (activation) and attenuation of H3K27me3 (repression) marks in non-NAB and NAB colonies, compared to un-induced limbal progenitors (F, G). Immunoprecipitation values were normalized to those obtained from ES cells. Examination of the efficiency of reprogramming by non-NAB and NAB methods, calculated by number of ALP positive colonies/number of cells plated (H), revealed 0.06% and 0.12%, respectively (I). Analysis of efficiency of non-NAB colony formation in the presence of complete ESCM, heat-treated ESCM and exosome depleted ESCM revealed statistically significant difference between the groups (*p<0.05; ***<p.0001; One tailed t test) (J). Comparison of ESCC miRNA (miR 294, miR 295) and miR 302 between exosomes derived from ESCM and MEFCM revealed upregulation of the miRNAs in the former than latter (K–M).

### Induction by the Non-NAB Approach

Mouse D3 ES cells (ATCC) were cultured in gelatin-coated flasks in the presence of 2000 units/ml of leukemia inhibitory factor (LIF). Embryonic stem cell conditioned medium (ESCM) was collected when cells were 60% confluent. The medium was centrifuged, passed through 0.22 µm filter and used either fresh or after storage at −80°C. Secondary limbal neurospheres were cultured in equal volumes of ESCM and DMEM F12, containing N2 supplement (1×), 2 mM Glutamine, and 1% FBS (1∶1) for the first 5 days. MAPK inhibitor (PD0325901;1 µM) (Stemgent) and GSK3β inhibitor (CHIR99021; 3 µM) (Stemgent) [12] were added to the medium and culturing was continued until the appearance of ES like colonies under feeder-free conditions. Controls included limbal neurospheres cultured without ESCM in the presence of small molecules, which did not yield any colony. To rule out trace mouse ES cells in the conditioned medium as a source of reprogrammed cells, GFP expressing limbal progenitors were cultured to distinguish between GFP^+^ and GFP^–^ (contaminant) colonies [13].

### Induction by the NAB Approach

STEMCCA lentiviruses were produced by transfecting the 293T packaging cells as previously described [19]. Supernatants containing viral particles were collected at 48 and 72 hours post transfection. Viral particles were concentrated hundred-fold using PEG virus precipitation kit (Biovision) following the manufacture’s protocol. Limbal cells (4×10^5^), trypsinised from secondary neurospheres, were seeded/well of six well plates, and infected with 10 µl of concentrated virus in the presence of polybrene (8 µg/ml). The medium was replaced after 16 hours with mouse embryonic stem (ES) cell medium (DMEM supplemented with 20% FBS, L-glutamine, nucleosides, β-mercaptoethanol, and 2,000 U/ml LIF), and changed on alternate days. Colonies were picked after 20 DIV post-infection and expanded by plating on mitomycin C-treated MEFs in ES cell medium.

### Generation of EBs

Embryoid bodies (EBs) were generated from non-NAB and NAB limbal iPS cells by hanging drop culture methods as previously described [13]. Briefly, cells were suspended in IMDM containing 20% FBS and cultured in 50 µl droplets ( = ∼100 cells/droplet) inside a lid of a sterile 100 mm Petri dish with PBS for 3 days at 37°C [20].

**Figure 3:**
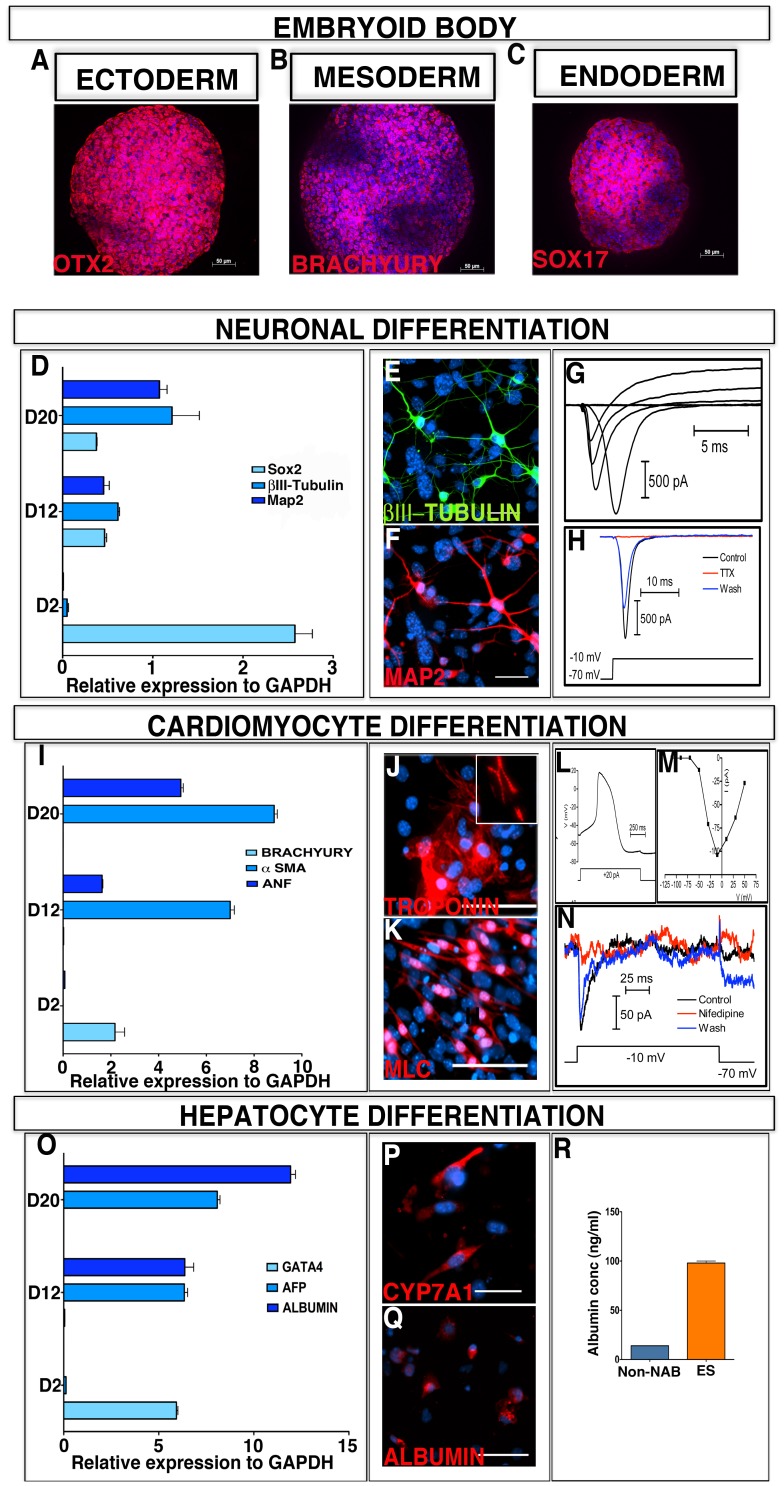
*In vitro* pluripotency of colonies generated by the non-NAB method. Non-NAB iPS colonies subjected to hanging drop culture generated EBs positive for Ectodermal (OTX2); Mesodermal (BRACHYURY) and Endodermal (SOX-17) markers (A–C). EBs thus generated were subjected to neuronal, cardiomyocytes, and hepatocyte differentiation protocols established for mouse ES cells. Q-PCR analysis of transcripts revealed temporally regulated differentiation of all three lineages (D, I, O). Cells at the end of neuronal differentiation phase expressed immunoreactivities corresponding to β*III-TUBULIN* (E) and MAP2 (F). Whole-cell recordings of these cells revealed fast-acting inward currents due to voltage-gated sodium channel (G), blocked by TTX (1 µM) (H), characteristics of neurons. Cells at the end of cardiomyocyte differentiation phase expressed immunoreactivities corresponding to TROPONIN (J) and MLC (K). Whole-cell recordings of beating cardiomyocytes revealed the presence of L-type calcium currents (L) blocked by nifedipine (5 µM) and action potentials characteristic of ventricular cardiomyocytes (M, N). Cells at the end of hepatocyte differentiation phase expressed immunoreactivities corresponding to CYP7A1 (P) and ALBUMIN (Q). Differentiated limbal iPS cells, like differentiated mouse ES cells, elaborated albumin into the culture medium, albeit at lower levels (R). Scale bar: E,F; J,K; P,Q 50 µm.

**Figure 4:**
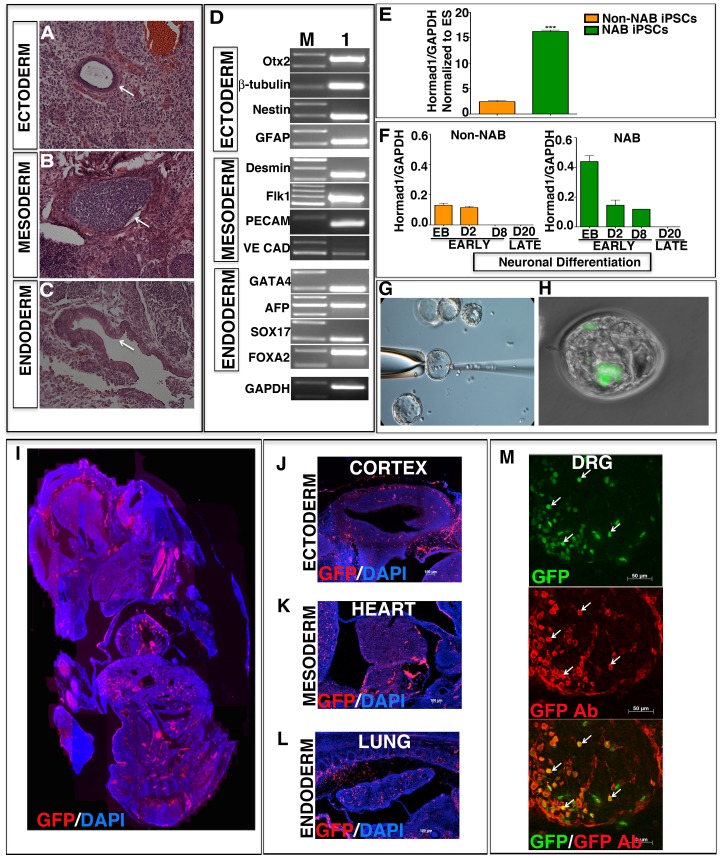
*In vivo* pluripotency of colonies generated by the non-NAB method. Cell dissociates from non-NAB colonies injected subcutaneously into NOD-SCID gamma chain knock out (NSG) mice formed teratomas that contained tissues of all three embryonic lineages; ectoderm (duct), mesoderm (cartilage), and endoderm (glandular columnar epithelium with brush border) (A–C). Examination of teratomas by RT-PCR analysis revealed the presence of transcripts corresponding to markers of embryonic ectoderm, mesoderm and endoderm (D). Q-PCR analyses of transcripts of *Hormad1* revealed significantly lower expression in limbal iPS cells, generated by the non-NAB method compared to the NAB method (E). Q-PCR analyses of *Hormad1* expression during neuronal differentiation, revealed the relative absence of *Hormad1* transcripts on day 8 in non-NAB cells, compared to NAB cells (F). Microinjection of GFP^+^ non-NAB limbal iPS cells into morulae (G), followed by their *in vitro* development revealed their integration into inner cell mass of an early blastocyst (H). A saggital section of an E14 chimeric embryo (I), obtained by blastocyst injection of GFP expressing iPS cells, reprogrammed by non-NAB method, revealed the incorporation of GFP^+^ cells in multiple developing tissues, including the cerebral cortex (ectoderm), heart (mesoderm), and lung (endoderm) (J–L). Co-localization of GFP with immunoreactivities to GFP antibody in dorsal root ganglion (DRG) cells validated the contribution of GFP^+^ cells to E14 chimeric embryo (M). Immunoreactivities corresponding to GFP were identified by immunohistochemistry using a primary antibody against GFP and CY3 conjugated secondary antibody in Figures (I–L). Lane M = marker; lane 1 = teratoma. The image in I represents a montage of multiple images assembled manually. The sizes of the PCR amplified products presented in panel D is provided in Table S1.

### Neuronal Differentiation

Non-NAB and NAB limbal iPS cells were differentiated into neurons by a previously described method [21]. Briefly, EBs generated from mouse limbal iPS cells were cultured in neural induction medium for 5–7 days at 37°C. The resulting cell clusters were manually triturated and plated on PDL/laminin coated dishes with neural expansion medium [neural induction medium +20 ng/ml of FGF2 (R&D Systems)] for 25 days.

### Cardiomyocyte Differentiation

Limbal iPS cells were differentiated into cardiomyocytes by a previously described method [20]. Briefly, EBs were plated on gelatin-coated dish and cultured in IMDM +20% FBS for 48 hours at 37°C. The medium was changed to IMDM +0.2% FBS and culture was maintained for 15 days.

### Hepatocyte Differentiation

Limbal iPS cells were differentiated into hepatocytes by a previously described method [22]. Briefly, EBs generated by limbal iPS cells were cultured in matrigel (BD Bioscience)-coated dish in differentiation medium I [DMEM/F12, 1% FBS, 1% nonessential amino acids, 1% nucleosides, 1% penicillin + streptomycin, 1% glutamic acid, 3% BSA, 100 ng/ml of FGF2, and 100 ng/ml of Activin A (R&D Systems)] for 3 days at 37°C. The medium was changed to differentiation medium II [DMEM/F12, 15% FBS, 1% nonessential amino acids, 1% nucleosides, 1% penicillin + streptomycin, 1% glutamic acid, 10 ng/ml of HGF (R&D Systems)] and culture was continued for 8 days at 37°C. The medium was changed to differentiation medium III [differentiation medium II +10^−7^ M of dexamethasone (Sigma)] and cells were cultured for another 10 days.

### Albumin Secretion

The culture supernatant was collected and stored at −20°C for analysis of albumin. Albumin estimation was performed according to the manufacturer’s protocol using mouse albumin ELISA kit (Immunology Consultants Laboratory, Inc. Newberg, OR).

### PCR Analysis

PCR analysis was performed as previously described [13]. Total RNA was extracted from cells using the MiniRNeasy Kit according to the manufacturer’s instructions. Complementary DNA synthesis was carried out on 5 µg of total RNA/sample using the SuperscriptIII RT kit (Invitrogen) following the manufacturer’s instructions. Transcripts were amplified and their levels quantified using gene-specific primers (Table S1) and Quantifast SYBR Green PCR kit (Qiagen) on a RotorGene 6,000 (Corbett Robotics, San Francisco, CA). Measurements were performed in triplicates; a reverse-transcription-negative blank of each sample and a no-template blank served as negative controls. Gene expression levels were normalized to the expression of the housekeeping gene GAPDH. The results obtained were analyzed by one tailed t test or ANOVA. A p value <0.05 was considered significant.

### Immunocytochemistry

Immunocytochemical analysis was carried out for the detection of cell-specific markers as previously described [13]. Briefly, paraformaldehyde-fixed cells were incubated in PBS containing 5% normal goat serum (NGS) and 0/0.2/0.4% Triton-X100 followed by overnight incubation in antibodies at 4°C. The list of antibodies is provided in Table S2. Cells were examined for epifluorescence following the incubation in IgG conjugated to Cy3/FITC. Images were acquired using a Zeiss ApoTome Imager M2 microscope (Axiovert 200 M) and captured by cooled CCD-camera (Zeiss). Axiovision 4.8 software was used for image processing.

### Microarray Analysis

Total RNA was isolated from the mouse ES cells, un-induced limbal progenitors, non-NAB, and NAB limbal iPS cells and used to synthesize biotin-labeled cRNA probe, using Gene Chip 3′ IVT Express kit (Affymetrix, Santa Clara, CA). Fragmented cRNA probes were hybridized to Mouse genome 430 2.0 Gene chip arrays (Affymetrix) at 45°C for 16 hours. The arrays were scanned using an Affymetrix GCS3000 7G device, and images were analyzed using the GCOS software. Normalization and expression values were calculated using log scale robust multiarray analysis, implemented in BioConductor.

### Alkaline Phosphatase Staining

Alkaline phosphatase staining was carried out using the Stemgent Alkaline phosphatase staining kit as per instructions.

### Bisulfite Genomic Sequencing

Bisulfite genomic sequencing was carried out on 0.36 µg of genomic DNA, using EZ DNA Methylation-Direct kit (Zymo Research, Orange, CA), according to the manufacturer’s instructions. Bisulfite modified DNA was amplified using gene-specific primers (Table S3) and cloned into TOPO vector (Invitrogen), and ten randomly selected clones were sequenced.

### Chromatin Immunoprecipitation (ChIP)

ChIP analysis was carried out as previously described [23]. Cells (1×10^7^) were cross-linked and serially quenched with 1% formaldehyde and glycine, respectively. Further processing was carried out using Chromatin Immunoprecipitation kit (Upstate) following the manufacturer’s protocol. Immunoprecipitation was carried out with anti-trimethyl histone 3 lysine 4 (H3K4me3, Abcam)/anti-trimethyl histone 3 lysine 27 (H3K27me3, Abcam). For controls, immunoprecipitation was carried out with specific IgG antibodies (Santa Cruz Biotechnology). The precipitated DNA was purified after proteinase and RNAse A digestion, using a Qiaquick PCR purification kit (Qiagen Inc). Q-PCR was carried out using a Quantifast SYBR Green PCR kit (Qiagen) on a RotorGene 6,000 (Corbett Robotics, San Francisco, CA). The primer sequences for the Q-PCR are provided in Table S4. The calculations were performed by percent input method and normalized to values obtained by ChIP analysis, carried out on the ES cells.

### Electrophysiological Analysis

Electrophysiological analysis was carried out as previously described [13]. Briefly, cells were plated on coverslips, placed in a chamber, and perfused on the stage of an upright, fixed-stage microscope (Olympus BHWI) with oxygenated Ames’ medium. Recordings were carried out at room temperature using patch pipettes (1–2 µm O.D. with tip resistances of 6–12), filled with a solution containing (in mM): KCH3SO4, 98; KCl, 44; NaCl, 3; HEPES, 5; EGTA, 3; MgCl2, 3; CaCl2, 1; glucose, 2; Mg-ATP, 1; GTP, 1 (pH 7.2). Recordings were obtained using an Axopatch 200B or Multiclamp amplifer (Axon Instruments, Molecular Devices, Sunnyvale, CA), and responses were acquired using a Digidata 1,322 interface and PClamp 9.2 software (Axon Instruments). Cells were voltage clamped at a steady membrane potential of −70 mV. Capacitative and leak currents were subtracted using a P/8 protocol.

### Generation of Teratomas

For teratoma induction, 2×10^6^ limbal iPS cells were injected subcutaneously into the dorsal flank of non-obese diabetic-severe combined immunodeficiency NOD-SCID gamma chain knockout (NSG) mice. Teratomas were recovered 3–4 weeks post injection, fixed overnight in 10% formalin, paraffin-embedded, and stained with Hematoxylin-eosin stain. Samples from teratomas were frozen for reverse transcription-PCR (RT-PCR) analysis.

### Blastocyst Injection and Generation of Chimeras

The generation of the chimeric mice was carried out with iPS cells generated from limbal progenitors of 129SvJ mouse strain (Nanog-GFP/ubiquitous eGFP positive) by standard procedures. Approximately, 8 to 10 iPS cells were injected into each C57BL/6J blastocyst cavity. Six to ten injected blastocysts were transferred to the uterus of pseudopregnant CD-1 females at 2.5 days post-fertilization. In the case of eGFP iPS cells the pregnant females were necropsied at embryonic day 14 and embryos were harvested. Sections were prepared and analyzed for GFP by immunofluorescence. In the case of Nanog GFP iPS cells, chimeric mice were allowed to grow full term and identified by coat color. These mice were crossed with C57BL/6J to detect germline transfer.

## Results

### Generation of Colonies with ES Cell Morphology

Neurospheres, representing the limbal epithelial progenitors generated in conditions of attenuated BMP signaling [18], were cultured in the presence of mouse ES cell conditioned medium (ESCM) for 20 days *in vitro* (DIV) (Figure. 1A). For comparing the reprogramming by non-NAB and NAB approaches, a parallel batch of neurospheres was similarly cultured without ESCM, following their transduction with polycistronic constitutive lentiviral vector STEMCCA to express *Oct4*, *Klf4*, *Sox2* and *c-Myc* (OKSM) simultaneously [19]. Controls included non-transduced neurospheres cultured without ESCM. In both cases the limbal progenitors generated colonies. In the case of the non-NAB approach, colonies (non-NAB colonies) could be routinely observed by 8±2 DIV (n = 11 observations), while in the NAB approach (NAB colonies) they were appreciated earlier, by 5±2 DIV (n = 4 observations). No colonies were detected in control cultures at 20DIV or later. In both approaches, the emergence of the colonies was either coincidental or temporally preceded by the activation of endogenous *Oct4* and *Nanog* genes (see below). These colonies, irrespective of the approaches to obtain them, acquired the morphology of mouse ES cell colonies by 20 DIV (Figure 1; B–D). They expressed immunoreactivities corresponding to pluripotency markers; OCT4, NANOG and SSEA1 like those derived from the ES cells (Figure. 1; E–M). A temporal analysis of pluripotency-related and cell-type specific gene expression, as an initial measure of reprogramming, detected transcripts corresponding to *Oct* and *Nanog* in non-NAB and NAB colonies at 8 and 4 DIV, respectively, the time when colonies first appeared (Figure. 1; N, P). The temporal activation of *Oct4* and *Nanog* genes was preceded by temporal attenuation in *p63* and *α-enolase* expression, suggesting that the reprogramming involved the reciprocal inhibition of the limbal specific genes (Figure. 1; O, Q). Both *p63* and *α-enolase* genes were completely silenced in non-NAB colonies by 8DIV. In contrast, such tight reciprocal temporal regulation of limbal-specific and pluripotency gene was lacking in NAB colonies. For example, after an initial decrease in the expression of *p63* by 4DIV, it reverted at 8DIV close to its initial levels and persisted, however without any bearing on the emergence of the colonies. The expression of limbal progenitor-specific genes in the starting population of cells and their progressive attenuation upon reprogramming ruled out extra-limbal contaminations. We had previously demonstrated a normal rat karyotype of non-NAB colonies when rat limbal progenitors were targeted for reprogramming thus ruling out the possibility of contaminant mouse ES cells in the conditioned medium as the source of the colonies [13]. Here, in a different approach to rule out the contamination, we subjected GFP-expressing mouse limbal progenitors to non-NAB reprogramming. The resulting colonies were all GFP-positive confirming that the sources of colonies were mouse limbal cells and not contaminant ES cells (Figure 1B (inset)). Together, these observations suggested that both non-NAB and NAB colonies displayed the morphological and biochemical phenotype of ES cell colonies.

### Changes in Global Gene Expression and Epigenetic Status

Next, we examined whether or not the acquired ES cell phenotype of non-NAB and NAB colonies was reflected in global gene expression patterns and epigenetic status characteristic of the ES cells. A comparison of transcriptional profiles by microarray analyses revealed a pattern of expression in non-NAB colonies that was distinctively different from un-induced limbal progenitors (*R^2^* = 0.55; p<0.0001) and similar to that of ES (*R^2^* = 0.93; p<0.0001) and NAB (*R^2^* = 0.93; p<0.0001) cells (Figure. 2 A–C). Both the non-NAB and NAB colonies shared the expression of a core group of genes, underlying the regulatory network of pluripotency [24]–[26], with ES cells. The expression of the majority (70%) of these pluripotency regulators was increased in the non-NAB and NAB colonies, compared to un-induced limbal progenitors, suggesting that the induction by both ESCM and exogenous TFs alters global gene expression that may favor the acquisition of pluripotency. The expression of the key pluripotency gene, *Oct4* and that of *Nanog*, *Lin28* and *Glis1*, which is known to facilitate Oct4-mediated reprogramming [27], was corroborated by Q-PCR analysis (Figure. 2D). Transcripts corresponding to these (except *Glis1*) and other regulatory genes (Figure S1) remained undetectable or at the base levels in un-induced limbal progenitors. In addition, a substantial increase in the expression of chromatin remodeling factor *Brg1* (*Smarca 4*), known to facilitate four-factor reprogramming, was seen in non-NAB and NAB colonies over uninduced limbal progenitors [26]. The expression of pluripotency genes showed good correlation between non-NAB and NAB iPSCs (R = 0.88). To determine whether or not the non-NAB and NAB cells have acquired an ES cell-like epigenetic signature, we first determined the comparative methylation status of CpGs dinucleotides in *Oct4* and *Nanog* promoters, which is an indicator of their relative activities. Bisulfite sequencing of these promoters in un-induced neurospheres revealed that they were hypo-methylated (*Oct4*, 10%; *Nanog*, 8.3%) to begin with, a reflection of their malleable nature at the molecular levels. However, the methylation status revealed a relatively decreasing trend in non-NAB cells (*Oct4*, 0%; *Nanog*, 5%) and NAB cells (*Oct4*, 7.3%; *Nanog*, 8.3%), the changes being more pronounced in the former and closer to ES cell levels (*Oct4*, 3.3%; *Nanog* 3.3%) than the latter (Figure. 2E). Next, to obtain another perspective on the epigenetic status of the induced cells, we compared the histone methylation patterns in *Oct4* and *Nanog* promoters in terms of H3K4 and H3K27 trimethylation, the former associated with active genes [28] and the latter with those that are silenced [29]. ChIP analysis revealed that *Oct4* and *Nanog* promoters in non-NAB and NAB cells, like ES cells, were characterized by H3K4me3 activation marks while those in un-induced cells by H3K27me3 repression marks (Figure. 2 F, G). The presence of low levels of H3K4me3 marks on the *Oct4* promoter in un-induced limbal progenitors with co-existing H3K27me3 marks may reflect transitory inductive changes as observed during the reprogramming of mouse embryonic fibroblasts; genes which have H3K27me3 marks before reprogramming start to acquire low levels of H3K4me3 marks [30] (Figure. 2G).The chromatin immunoprecipitation results between between non-NAB and NAB iPSCs showed a good correlation (R = 0.96). Together, these observations suggested that the non-NAB and NAB cells acquired an epigenetic status similar to that of ES cells resulting in comparable global gene expression patterns that included the expression of pluripotency network genes.

### Reprogramming Efficiency and Non-cell Autonomous Influence

Next, we examined the relative reprogramming efficiency of non-NAB and NAB approaches. Quantification of the colony forming efficiency, based on the emergence of alkaline phosphatase (ALP) colonies from total cells plated at 20 DIV, revealed the efficiency for non-NAB and NAB approaches to be 0.0625% and 0.12%, respectively (Figure. 2 H, I). The difference in non-NAB and NAB colonies extended to their ability for passaging; while the latter can be readily passaged and single cell cloned, the former demonstrated limited passaging ability and senesced after 6 passages. This difference in passaging ability may be attributed to differential expression pattern of *P63* gene. Given the observation that *p63* endows cell survival on epithelial cells [31] the persistence of *p63* expression might have allowed NAB colonies to overcome senescence necessary for passaging, that non-NAB colonies could not in its absence. Next, we were interested in defining the nature of the re-programming activities in the ESCM based on the premise that these could have peptide and/or nucleotide backbone, the exchange of the latter likely to be facilitated by exosomes [32]. We observed that the denaturation of proteins by heat treatment of the ESCM completely abolished colony formation, whereas colonies were formed when cells were cultured in exosome-depleted ESCM (Figure. 2J). However, the number of colonies was significantly reduced compared to controls. This observation suggested that the reprogramming activities lie in the protein fraction of the ESCM, and its efficiency may be influenced by exosomes. Exosomes have been observed to contain miRNA [32], and miRNA have been shown to improve the efficiency of reprogramming. For example, miR294 and miR295 increase the efficiency of TF-based reprogramming [33] while miR302 and miR367 have been demonstrated to reprogram fibroblasts without exogenous TFs [34]. To know whether these miRNAs might play a role in exosome-mediated regulation of reprogramming we screened exosomes isolated from ESCM for miRNAs; exosomes isolated from mouse embryonic fibroblast conditioned medium (MEFCM) were screened as controls. All miRNAs, known for their reprogramming properties except miR367, were present in ESCM exosomes and not in MEFCM exosomes (Figure. 2K–M). Let-7 miRNA expression, examined as a constitutive control, was present in both ESCM and MEFCM exosomes (data not shown). These observations suggested that the efficiency of reprogramming by ESCM might be facilitated by exosomal miRNA in non-NAB approach.

### Differentiation Along the Germ Lineages *in vitro* and *in vivo*

Next, we examined whether or not the reprogramming by non-NAB and NAB approaches had led cells to acquire the potential to generate differentiated cells of the three embryonic lineages. Since the burden of proof of pluripotency was much more on the non-NAB cells than those derived by the conventional NAB approach, the pluripotency of the former is discussed in detail in the backdrop of the latter (supporting information). When non-NAB (Figure. 3A–C) and NAB cells (Figure. S2A–C) were subjected to the conventional hanging drop culture [20] they generated embryoid bodies (EBs) at the same time (5 DIV), of the same size (150–200 µM), and expressing three germ layer markers as the ES cells. When subjected to directed neural differentiation protocol for ES cells [21], non-NAB cells acquired typical neuronal morphology, elaborated immunoreactivities corresponding to βIII-tubulin and Map2 (Figure. 3 E,F), and displayed electrophysiological signature of functional neurons, i.e., TTX-sensitive voltage-gated sodium currents (Figure. 3 G,H). Similarly, when subjected to a directed cardiomyocyte differentiation protocol for the ES cells [20] non-NAB cells differentiated into beating cardiomyocytes (video S1; NAB cardiomyocytes –video S2), displaying typical cardiomyocyte morphology with sarcomeric appearance and immunoreactivities corresponding to Troponin and Myosin light chains (MLC) (Figure. 3 J,K). The beating cardiomyocytes displayed voltage-sensitive L type calcium channel blocked by nifedipine, and lengthy action potentials, characteristic of ventricular cardiomyocytes (Figure. 3 L–N). Non-NAB cells were also capable of differentiating along the endodermal lineage; when subjected to a directed hepatocyte differentiation protocol [22] they displayed immunoreactivities of mature hepatocytes, Cyp7A1 and expressed and elaborated albumin (Figure. 3 P,Q) as ES cell-derived hepatocytes, albeit at different levels, suggesting their differentiation to hepatocytes (Figure. 3R). In each of the cases, the differentiation along a particular lineage was temporally regulated; the expression of mature markers [Map2 (neuronal); ANF (cardiomyocytes); albumin (hepatocyte)] was preceded by the lineage-specific progenitor markers [Sox2 (neuronal); Brachyury (cardiomyocytes); GATA4 (hepatocyte)] (Figure. 3 D, I, O). A similar differentiation potential along three germ lines was observed in NAB iPS cells (Figure. S2 D–L). Next, the pluripotency of limbal iPS cells was tested *in vivo*. First, un-induced limbal progenitors non-NAB limbal, and NAB limbal iPS cells were injected in NSG mice to generate teratomas. NSG mice injected with limbal iPS cells developed teratoma by four weeks while none were observed in mice injected with un-induced cells. Histological examination of teratomas revealed the presence of tissues belonging to all three-germ lineages; ductal (ectoderm), cartilaginous (mesoderm) and glandular (endoderm) (Figure. 4 A–C: Non-NAB iPS cells; Figure. S3 A–C: NAB iPS cells). Further examination of teratoma for lineage specific genes by RT-PCR revealed the presence of transcripts corresponding embryonic ectoderm, mesoderm, and endoderm specific genes (Figure. 4D: Non-NAB iPS cells; Figure. S3 D: NAB iPS cells). Given the recent report that the teratomas generated by iPS cells using transitory episomal vectors are less immunogenic than those using retroviral vectors we compared the expression of genes associated with immunogenic responses of iPS cell-dependent teratomas [2]. The expression of *Hormad1*, one of three genes, was significantly lower in non-NAB than in NAB limbal iPS cells (Figure. 4E), suggesting that non-cell autonomously derived cells may be less immunogenic than those derived using viral vectors. The expression of other two genes, *Zg16* and *Cyp3a11*, were not detected in both non-NAB and NAB cells. Given the propensity of iPS cells for teratoma formation it is likely that lineage-committed post-mitotic precursors of these cells will be preferred for cell therapy. Therefore, we examined the temporal expression pattern of *Hormad1* during early and late stages of neuronal differentiation of non-NAB and NAB cells *in vitro* (Figure 4F). We observed that *Hormad1* expression during the early stages of differentiation (EBs to Day 8 in culture), when the majority of committed precursors are likely to be generated, was significantly lower in non-NAB cells than NAB cells. By day 8, while *Hormad1* expression persisted in the latter, it was undetectable in the former, suggesting that non-NAB cell-derived precursors are likely to be less immunogenic than their NAB counterparts. At the late stage, characterized by fully differentiated neurons (Figure 3E–H; Figure S2 D, E), *Hormad1* expression was undetectable in both non-NAB and NAB cells (Figure 4F). Second, GFP positive iPS cells from 129SvJ mice were injected into C57BL/6J mice blastocysts to determine the *in vivo* contributions of these cells to germ lineages. Blastocysts injected with GFP cells (Figure. 4 G, H), transferred into surrogate females, led to the development of chimeric embryos (Figure. 4I). A robust and widespread contribution of GFP cells was observed, particularly in the brain, heart, and lungs of the mid-gestational embryos (Figure. 4J–L). Co-localization of GFP with immunoreactivities to GFP antibody in dorsal root ganglion (DRG) cells validated the contribution of GFP cells to E14 chimeric embryos (Figure 4M). Though chimeric pups were born using either non-NAB or NAB limbal iPS cells (Figure. S3 E–I) albeit, with different levels of coat color contribution and bred, germ line transmission was not observed. The efficiency of chimerism was 9.5% and 12.5% for non-NAB and NAB reprogramming, respectively. Together, these observations demonstrated that limbal progenitors could be reprogrammed to a pluripotent state, capable of tri-lineage differentiation *in vitro* and *in vivo*.

## Discussion

We carried out a comparative analysis of reprogramming by non-NAB and NAB approaches to validate the proof of principle of a simple reprogramming of somatic progenitors under the inductive influence of ES cells [13], [35]. Reprogramming induced by the non-NAB approach is comparable to that achieved by NAB approach in terms of the de-novo activation of *Oct4* and *Nanog,* and emergence of colonies, similar to those generated by ES cells. Although the efficiency of colony formation with the NAB approach was 2 fold greater than the non-NAB approach, that achieved by the latter, was significantly better than previously reported re-programming by the NAB approach of using exogenous TFs [35]. The activation of *Oct4* and *Nanog* and the accompanied attenuation of *p63* and *α-enolase*, preceding the emergence of pluripotent colonies, were tightly regulated in non-NAB cells, while such a temporal and reciprocal expression pattern was not observed in NAB cells. Such differences in temporal and reciprocal expression pattern could be attributed to different mechanisms by which the two approaches are likely to influence the genome of the target cells; the non-NAB approach recruits the cells’ signal transduction machinery whose effects on the genome are likely to be nuanced versus the NAB approach where exogenous TFs promote gene expression, which is less calibrated in the absence of a defined ratio of ectopically expressed factors. The importance of the difference in the pattern of gene regulation on pluripotency is not immediately apparent as the indices of reprogramming in both cell types appear comparable but it could be speculated that unregulated expression pattern may underlie increased expression of *Hormad1* and incomplete silencing of *p63* in NAB cells. The latter could explain the relative lack of senescence observed in NAB cells, compared to the non-NAB limbal iPS cells. For example, *p63*, which is known to endow cell survival potential on epithelial cells, is likely to be protective against apoptosis in NAB cells while its absence in non-NAB cells may lead to their premature senescence [31]. Additionally, it is possible that the inability of the non-NAB approach to inhibit the expression of *p53*, a gene associated with cell cycle arrest, apoptosis and senescence, may underlie poor passaging and/or senescence of non-NAB cells [36]. Although a similar *p53* transcript levels in non-NAB and NAB cells suggests otherwise (Figure S4) an extensive examination of p53 expression at transcriptional and post-translational levels is needed before ruling out its involvement. Both non-NAB and NAB cells were comparable in their pluripotency in generating embryoid bodies, expressing early lineage markers, *in vitro* differentiation into cells of three germ lineages, and chimera formation. Our limited attempts at chimera generation did not result in true germ line transmission, despite a relatively high contribution of the non-NAB cells to other germ layers (Figure. 4I–M) consistent with their ability to differentiate into functional derivatives of these germ layers *in vitro* (Figure. 3) and high coat color contribution by NAB cells (Figure S3H). Our data do not allow us to attribute this failure to any specific difference between these pluripotent cells. The two known predictors of the quality of iPS cells, *Nanog* [37] and *Tbx3* [25] are expressed in limbal iPS cells (Figure. 2D; Figure S1). Given the observations that the frequency of germ line competence of the iPS cells is generally low [25] and quite variable, even in *Nanog* [37] and *Tbx3* [25] iPS clones, the apparent absence here likely reflects the associated low frequency and variability rather than the quality of the limbal iPS cells.

The non-cell autonomous reprogramming demonstrated here invokes the influence of the environment on the target cells, which are metastable. The metastable status of the limbal progenitors are characterized by (1) the prior expression of all Yamanaka reprogramming factors [13] except Oct4 and recently identified Glis1 [27], and (2) hypo-methylation status of *Oct4* and *Nanog* genes, which may have made these cells malleable to non-cell autonomous reprogramming. Additionally, the epithelial nature of the progenitors may add to this advantage, unburdening the process of additional steps required for mesenchymal to epithelial transition (MET) [38], [39]. Based on this logic we predict that stem cells/progenitors of epithelial nature, with prior expression of some of the pluripotency genes, will be more conducive to non-cell autonomous reprogramming than other somatic cells. For example, adult neural stem cells that express SOX2, cMYC, KLF4, and SSEA1 [10] may represent such suitable cell types. The mechanism of ES cell-mediated induction of pluripotency in limbal progenitors remains to be elucidated. It is likely to include soluble ligands activating intercellular signaling pathways influencing the network of pluripotency genes [35]. In addition, the involvement of ES cell cycle (ESCC)-specific miRNAs, which are observed to regulate ES cell self-renewal [40], reprogram human fibroblasts [6], and can be potentially imported via exosomes in the ESCM, is worth consideration. The advantage of the non-NAB approach to reprogramming is the regulated induction of pluripotency genes, without the concern of insertional mutagenesis associated with ectopic expression of exogenous TFs and the possibility of increasing the efficiency in conjunction with small molecules, once the induction pathways are identified. Additionally, the significant low level expression of *Hormad1*, a gene associated with immunogenic responses to iPS cells, in non-NAB limbal iPS cells [2], compared to NAB counterparts, suggests that iPS cells derived non-cell autonomously may be more suitable for autologous cell therapy by potentially eliciting either low or no immunogenic responses.

### Conclusions

Our analysis posits the non-NAB approach as a simple and viable method for reprogramming adult somatic progenitors, comparable to the NAB approach. This approach likely owes its success to the metastable status of progenitors of epithelial nature as demonstrated here by the limbal progenitors, which have been successfully used in stem cell therapy to treat blindness [17]. Reprogramming limbal progenitors to pluripotency by the non-cell autonomous technology, using conditioned medium as described here or through small moleclues, widens the scope of these easily accessible and malleable cells for safe and practical autologous cell therapy and for understanding disease processes beyond eyes.

## Supporting Information

Figure S1 **Analysis of transcripts of pluripotency.** Q-PCR analyses of transcripts corresponding to selected genes under the regulatory network of pluripotency revealed their levels comparable in non-NAB and NAB colonies but undetectable in un-induced cells (inverted arrows). Levels of transcripts are normalized to those in ES cells.
(TIF)

Figure S2 ***In vitro*** **differentiation of NAB iPS cells.** Limbal iPS cells generated by the non-NAB method subjected to hanging drop culture generated embryoid bodies expressing immunoreactivities to ectoderm (OTX2), Mesoderm (BRACHYURY) and Endoderm (SOX-17) (A–C). Neurally induced NAB cells revealed expression of neuronal markers βIII-TUBULIN (D), MAP2 (E). RT-PCR analysis revealed the expression of transcripts corresponding to neuronal regulator, Mash1, and markers, βIII-tubulin and Map2 (F). Cells induced along the cardiomyocyte lineage revealed the expression of mature markers TROPONIN (G) and MYOSIN LIGHT CHAIN (MLC) (H). RT-PCR analysis revealed the expression of transcripts corresponding to cardiomyocyte markers, αSMA, α−MHC, and β−MHC (I). Cells induced towards the hepatocyte lineage revealed expression of mature markers ALBUMIN (J) and CYP7A1 (K). RT-PCR analysis revealed the expression of transcripts corresponding to hepatocyte markers, *Aldolase B, Albumin* and *Cyp7a1* (L). Lanes: M = Marker; N = Neurons; C = Cardiomyocytes; H = Hepatocytes. Scale bar: D,E; G,H; J,K 50 µm.The sizes of the PCR amplified products presented in panels F, I and L are provided in Table S1.
(TIF)

Figure S3 **Analysis of pluripotency by teratoma and chimera generation.** Cells dissociated from NAB colonies injected subcutaneously in NOD-SCID gamma chain knockout (NSG) mice formed teratomas that contained tissues of all three embryonic lineages; ectoderm (duct), mesoderm (immature cartilage), and endoderm (glandular columnar epithelium with brush border) (A–C). Examination of teratomas by RT-PCR analysis revealed the presence of transcripts corresponding to markers of embryonic ectoderm, mesoderm and endoderm (D). Chimeric mice were generated from both non-NAB (F) and NAB iPS cells (H) and compared with respective wild type controls (E, G). The contribution of non-NAB iPS cells to coat color in the chimeric mice is demarcated by broken lines (F) and further confirmed by genotype analysis, which revealed the presence of the genomic sequence corresponding to GFP in non-NAB iPS chimera but not in the wild type control (I). The sizes of the amplified products represented in panels D and I are provided in Table S1.
(TIF)

Figure S4 **Analysis of p53 expression in non-NAB and NAB iPS colonies.** Q-PCR analysis of *p53* transcripts revealed no significant (p = 0.2895) difference between non-NAB and NAB iPS colonies.
(TIF)

Table S1 **List of gene specific primers.**
(DOC)

Table S2 **List of antibodies.**
(DOC)

Table S3 **List of primers for Bisulfite Sequencing.**
(DOC)

Table S4 **List of primers for Chromatin Immunoprecipitation.**
(DOC)

Video S1 **Beating cardiomyocytes differentiated from non-NAB iPS cells.**
(MP4)

Video S2 **Beating cardiomyocytes differentiated from NAB iPS cells.**
(MP4)
