# Supplementary figures and images for: Nucleic Acid and Non-Nucleic Acid-Based Reprogramming of Adult Limbal Progenitors to Pluripotency
Source: PLoS One. 2012 Oct 8;7(10):e46734. doi: 10.1371/journal.pone.0046734 (PMC3466310; doi:10.1371/journal.pone.0046734)

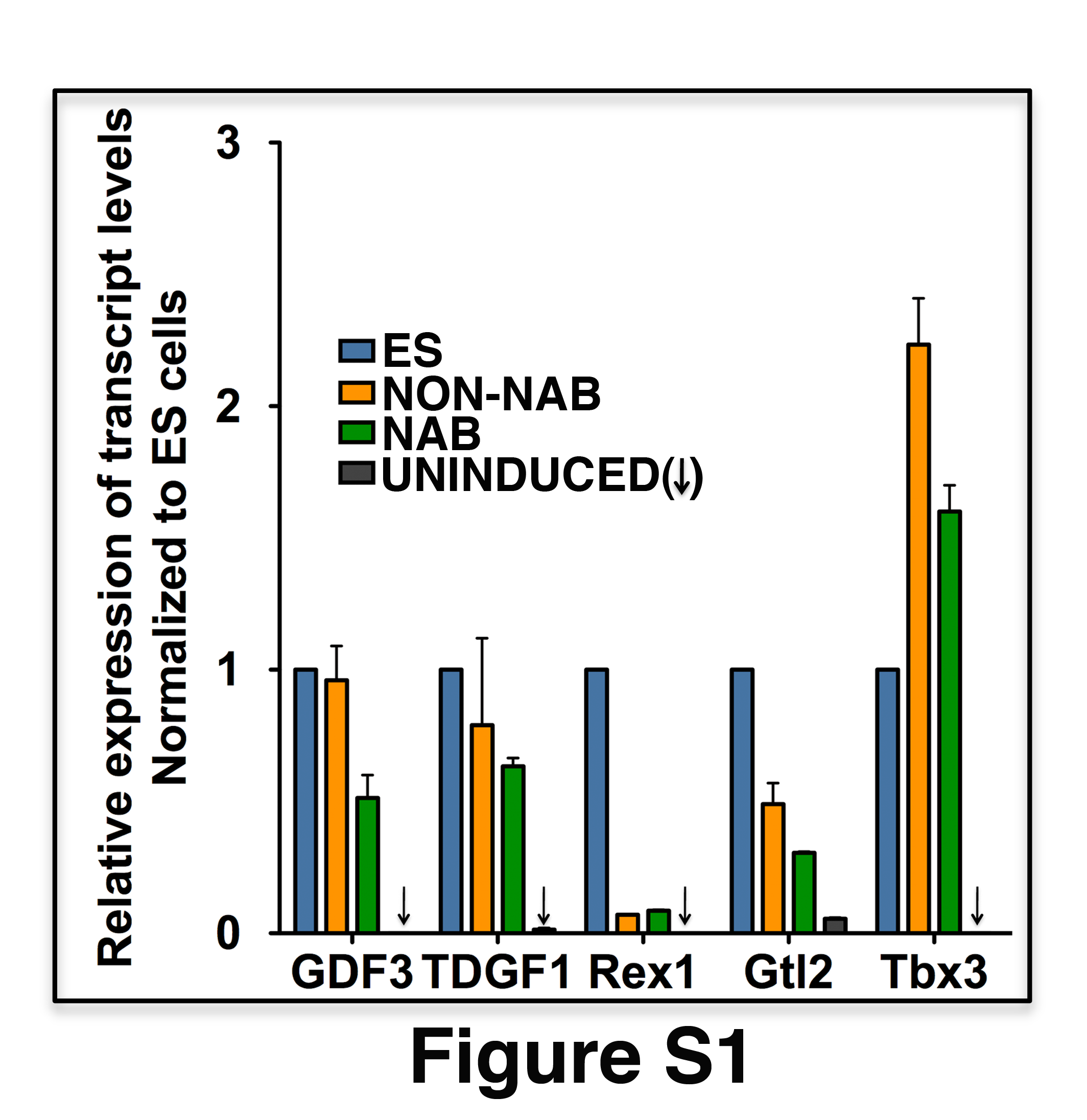

Supplement: Figure S1 — Analysis of transcripts of pluripotency. Q-PCR analyses of transcripts corresponding to selected genes under the regulatory network of pluripotency revealed their levels comparable in non-NAB and NAB colonies but undetectable in un-induced cells (inverted arrows). Levels of transcripts are normalized to those in ES cells. (TIF) [file pone.0046734.s001.tif]

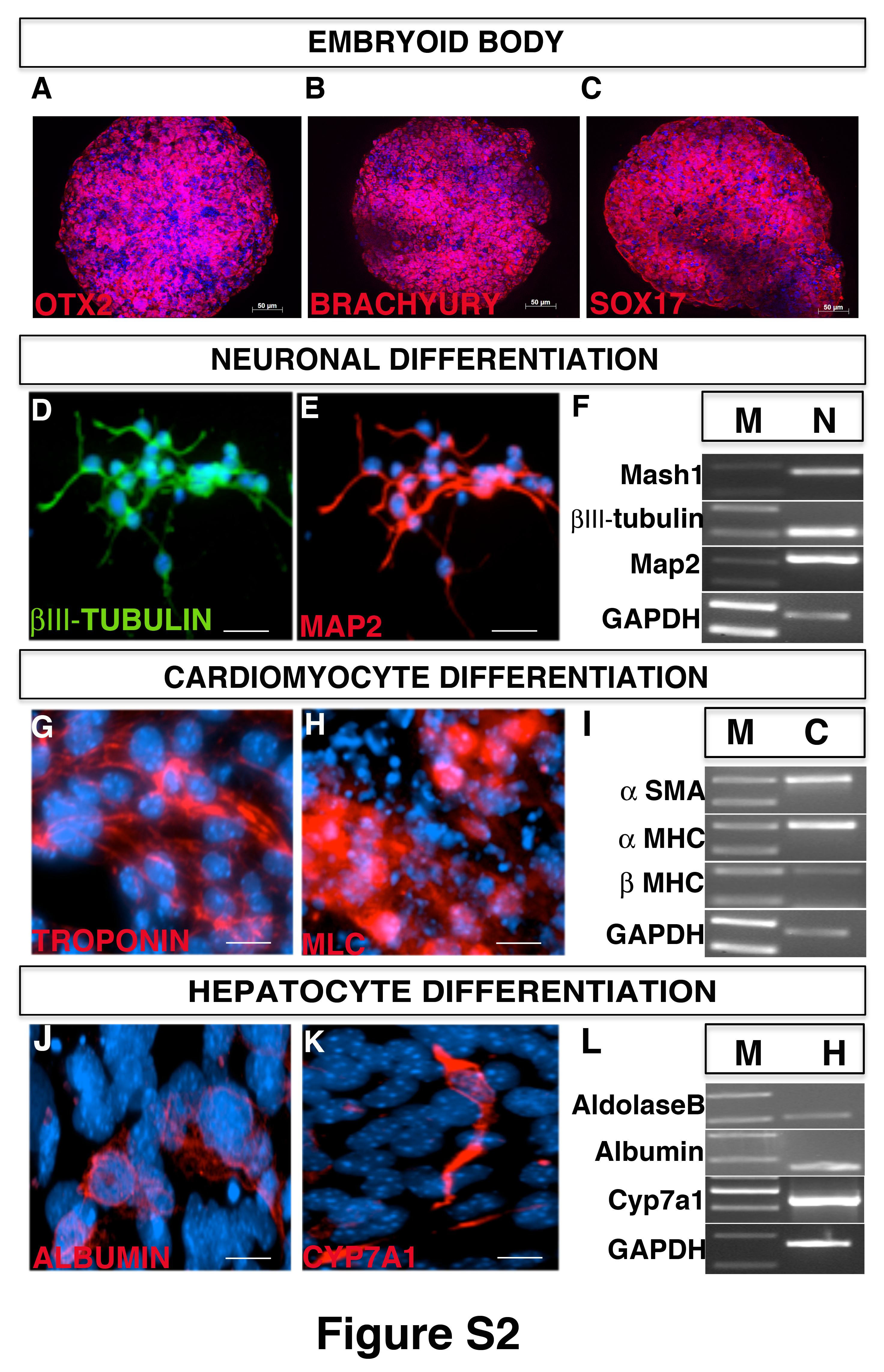

Supplement: Figure S2 — In vitro differentiation of NAB iPS cells. Limbal iPS cells generated by the non-NAB method subjected to hanging drop culture generated embryoid bodies expressing immunoreactivities to ectoderm (OTX2), Mesoderm (BRACHYURY) and Endoderm (SOX-17) (A–C). Neurally induced NAB cells revealed expression of neuronal markers βIII-TUBULIN (D), MAP2 (E). RT-PCR analysis revealed the expression of transcripts corresponding to neuronal regulator, Mash1, and markers, βIII-tubulin and Map2 (F). Cells induced along the cardiomyocyte lineage revealed the expression of mature markers TROPONIN (G) and MYOSIN LIGHT CHAIN (MLC) (H). RT-PCR analysis revealed the expression of transcripts corresponding to cardiomyocyte markers, αSMA, α−MHC, and β−MHC (I). Cells induced towards the hepatocyte lineage revealed expression of mature markers ALBUMIN (J) and CYP7A1 (K). RT-PCR analysis revealed the expression of transcripts corresponding to hepatocyte markers, Aldolase B, Albumin and Cyp7a1 (L). Lanes: M = Marker; N = Neurons; C = Cardiomyocytes; H = Hepatocytes. Scale bar: D,E; G,H; J,K 50 µm.The sizes of the PCR amplified products presented in panels F, I and L are provided in Table S1. (TIF) [file pone.0046734.s002.tif]

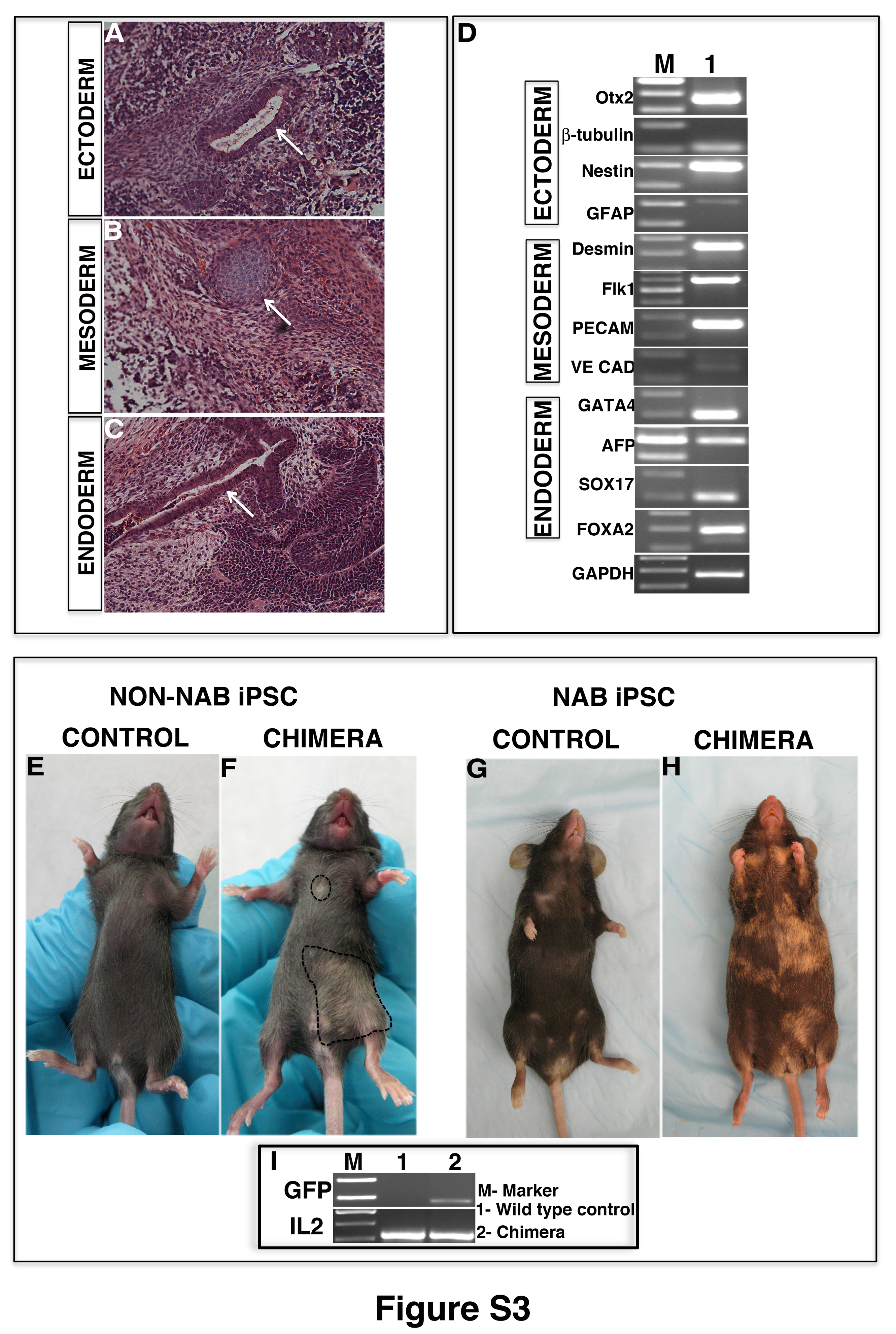

Supplement: Figure S3 — Analysis of pluripotency by teratoma and chimera generation. Cells dissociated from NAB colonies injected subcutaneously in NOD-SCID gamma chain knockout (NSG) mice formed teratomas that contained tissues of all three embryonic lineages; ectoderm (duct), mesoderm (immature cartilage), and endoderm (glandular columnar epithelium with brush border) (A–C). Examination of teratomas by RT-PCR analysis revealed the presence of transcripts corresponding to markers of embryonic ectoderm, mesoderm and endoderm (D). Chimeric mice were generated from both non-NAB (F) and NAB iPS cells (H) and compared with respective wild type controls (E, G). The contribution of non-NAB iPS cells to coat color in the chimeric mice is demarcated by broken lines (F) and further confirmed by genotype analysis, which revealed the presence of the genomic sequence corresponding to GFP in non-NAB iPS chimera but not in the wild type control (I). The sizes of the amplified products represented in panels D and I are provided in Table S1. (TIF) [file pone.0046734.s003.tif]

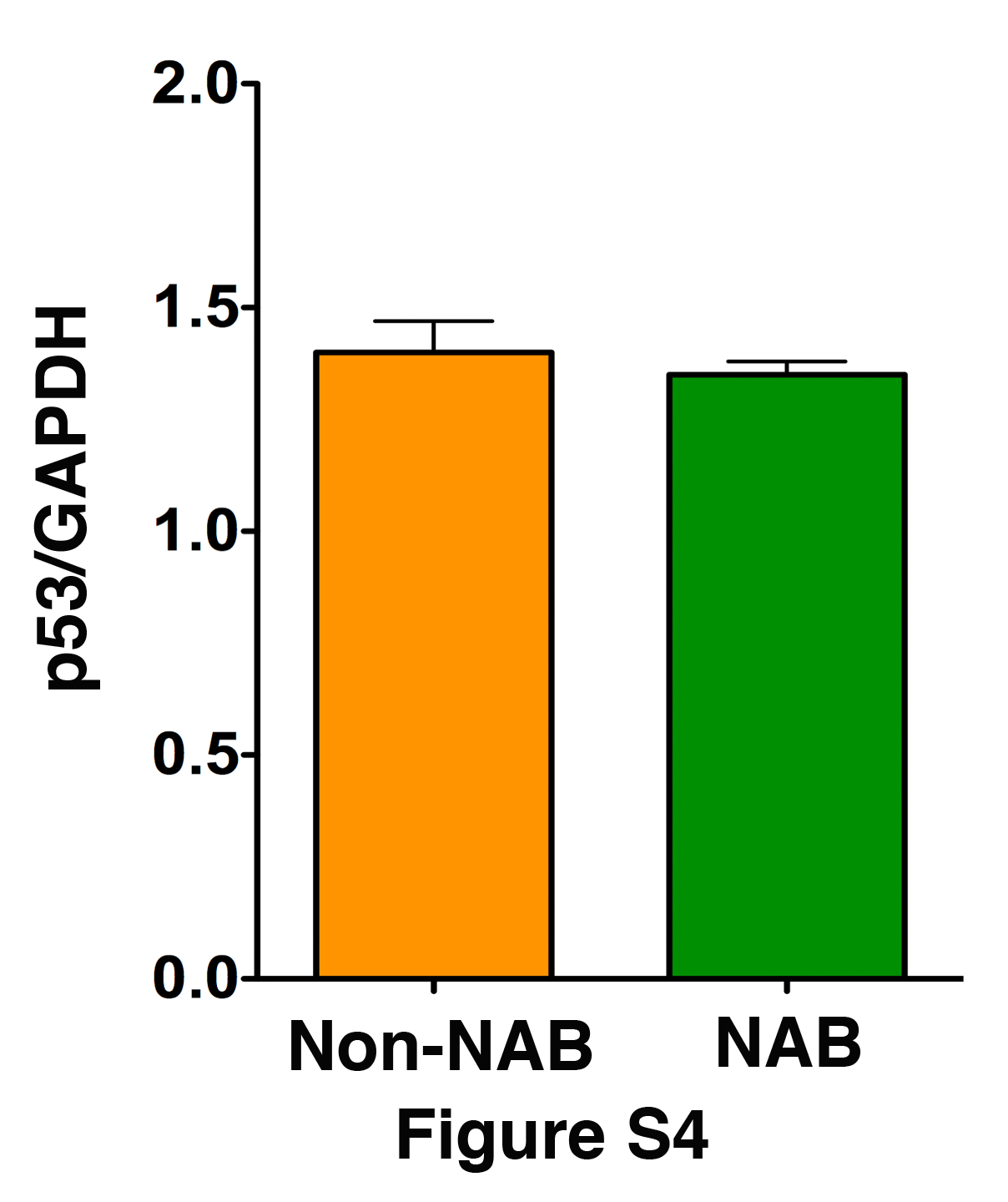

Supplement: Figure S4 — Analysis of p53 expression in non-NAB and NAB iPS colonies. Q-PCR analysis of p53 transcripts revealed no significant (p = 0.2895) difference between non-NAB and NAB iPS colonies. (TIF) [file pone.0046734.s004.tif]
