# Supplementary material for: Nucleic Acid and Non-Nucleic Acid-Based Reprogramming of Adult Limbal Progenitors to Pluripotency
Source: PLoS One. 2012 Oct 8;7(10):e46734. doi: 10.1371/journal.pone.0046734 (PMC3466310; doi:10.1371/journal.pone.0046734)
Supplement: Table S1 — List of gene specific primers. (DOC) [file pone.0046734.s005.doc]

Table S1: List of gene specific primers

| **Gene**  **name** | **Primer sequence** | **Annealing temperature** | **Product size** | **Accession No.** |
| --- | --- | --- | --- | --- |
| GAPDH | ACAGTCCATGCCATCACTGCC  GCCTGCTTCACCACCTTCTTG | 60 | 266 | NM_017008 |
| Alpha p63 | CACAGGCAGCTGCACGACT  TCATTCTCCTTCCTCTTTGATACGC | 56 | 234 | NM_011641 |
| Alpha enolase | TCCGAGACAATGATAAGACC  AAACCTCTGCTCCAATGC | 56 | 420 | NM_001025388 |
| Oct 4 | TCCCAACGAGAAGAGTATGAGGCC  CCGAGTAGAGTGTGGTGAAATGG | 58 | 130 | NM_001009178 |
| Nanog | GCAAGCGGTGGCAGAAAAAC  TATGGAGCGGAGCAGCATTC | 58 | 297 | NM_028016 |
| Lin 28 | CAGGGTTATTCTTTGGCTAATGGG  TCCTTTGTCTCATCCTTTCCTTCC | 57 | 174 | NM_145833 |
| Glis1 | TGCCACCAGGGTCTGTTTCTTC  TTGCTTGATGTGCTGAGCGG | 59 | 159 | NM_147221 |
| Brg1 | TGCCTGATGACCCACGATAC  TCTGTTTTGCTGCCCCAAG | 56 | 273 | NM_001174078 |
| Rex1 | ATCGCATCGCTGTGGGCATTAG  TTTGGGGACAACACTTGGAGGCAG | 57 | 168 | NM_009556 |
| GDF3 | ATTCACACTTGATTACCTCCCAGG  AACAGAAGGAAGCCGAGAGTCAG | 57 | 138 | NM_008108 |
| TDGF1 | GCCATCAGAGATAACAGCATTTGG  GTCCCTCCATTCAGACAGCAAG | 54 | 131 | NM_008108 |
| Gtl2 | TTGCACATTTCCTGTGGGAC  AAGCACCATGAGCCACTAGG | 57 | 288 | XR_035484 |
| Tbx3 | GGTGCTCTGGGCTGGATAAAAAG  TTTGGCATTTCGGGGTCTGC | 53 | 130 | NM_011535 |
| Otx2 | GCATAGAAGAAAAAGGAAGGGG  AATCAGTCGCACAATCCACACAG | 56 | 366 | AK087527 |
| Pax6 | TGGTGGTGTCTTTGTCAACGGG  TGGAGCCAGTCTCGTAATACCTGC | 58 | 180 | NM_013627 |
| Brachyury | CATGTACTCTTTCTTGCTGG  GGTCTCGGGAAAGCAGTGGC | 58 | 313 | NM_009309 |
| GATA4 | GGATTCAAACCAGAAAACGGAAGC  TGCCCATAGTGAGATGACAGCC | 58 | 190 | NM_008092 |
| Sox17 | CGATGAACGCCTTTATGGTGTG  TACTTGTAGTTGGGGTGGTCCTGC | 60 | 199 | NM_011441 |
| AFP | AGTGCGTGACGGAGAAGAAT  TGTCTGGAAGCACTCCTCCT | 58 | 494 | NM_007423 |
| Sox2 | AGGGCTGGGAGAAAGAAGAG  GGAGAATAGTTGGGGGGAAG | 56 | 177 | NM_011443 |
| Beta III tubulin | CTTTATCTTCGGTCAGAGTGGTGC  TTCTTTCCTCACGACATCCAGG | 55 | 103 | NM_023279 |
| Alpha SMA | GGCTGTTTTCCCATCCATCG  TGAGTCACACCATCTCCAGAGTCC | 58 | 399 | NM_007392 |
| ANF | AGGATTGGAGCCCAGAGTGGACTAGG  TGATAGATGAAGGCAGGAAGCCGC | 64 | 203 | NM_008725 |
| Albumin | TCTGGCACAATGAAGTGGGTAAC  GATACTGGGAAAAGGCAATCAGG | 55 | 172 | NM_009654 |
| Nestin | TGGAGCAGGAGAAGCAAGGTCTAC  TCAAGGGTATTAGGCAAGGGGG | 56 | 295 | NM_012987 |
| GFAP | ATCTGGAGAGGAAGGTTGAGTCG  TGGCGGCGATAGTCATTAGA | 58 | 310 | NM017009 |
| Desmin | CCTACACCTGCGAGATTGATGC  TTTGGTATGGACTTCAGAACCCC | 61 | 341 | NM_010043 |
| Flk1 | CCTGGTCAAACAGCTCATCA  AAGCGTCTGCCTCAATCACT | 57 | 599 | NM_010612 |
| PECAM | GTCATGGCCATGGTCGAGTA  CTCCTCGGCATCTTGCTGAA | 59 | 260 | NM_001032378 |
| VE cadherin | CCTGACTGGAACCAGCACGCT  GTGTGTCGTATGGGGGGCCAC | 64 | 491 | NM_009868 |
| FoxA2 | TGGTCACTGGGGACAAGGGAA  GCAACAACAGCAATAGAGAAC | 56 | 289 | NM_010446 |
| WPRE | TTCGCTTTCCCCCTCCCTATTG  CAGAATCCAGGTGGCAACACAG | 59 | 162 | Vector sequence |
| Mash1 | CCAACAAGAAGATGAGCAAGGTG  ACCCGCCATAGAGTTCAAGTCG | 59 | 170 | NM_008553 |
| Cyp7a1 | AGGACTTCACTCTACACC  GCAGTCGTTACATCATCC | 56 | 453 | NM_007824 |
| Beta MHC | GCCAACACCAACCTGTCCAAGTTC  TGCAAAGGCTCCAGGTCTGAGGGC | 64 | 205 | NM_010856 |
| Alpha MHC | GGAAGAGTGAGCGGCGCATCAAGG  CTGCTGGAGAGGTTATTCCTCG | 64 | 301 | NM_010856 |
| Aldolase B | TCTCCGTCAGGAAGCACCTC  ATGGCCTCTCTGAACGCTGT | 60 | 202 | NM_144903 |
| GFP | AAGTTCATCTGCACCACC  TCCTTGAAGAAGATGGTGCG | 60 | 176 |  |
| IL2 | CTAGGCCACAGAATTGAAAGATCT  GTAGGTGGAAATTCTAGCATCATCC | 60 | 324 | AF195956 |
