# Supplementary material for: Nucleic Acid and Non-Nucleic Acid-Based Reprogramming of Adult Limbal Progenitors to Pluripotency
Source: PLoS One. 2012 Oct 8;7(10):e46734. doi: 10.1371/journal.pone.0046734 (PMC3466310; doi:10.1371/journal.pone.0046734)
Supplement: Table S2 — List of antibodies. (DOC) [file pone.0046734.s006.doc]

**Table S2: List of antibodies**

| **Name** | **Species** | **Dilution** | **Company** |
| --- | --- | --- | --- |
| Oct4 | Rat | 1:100 | R & D systems |
| Nanog | Goat | 1:100 | R & D systems |
| SSEA1 | Rabbit | 1:100 | Abcam |
| Otx2 | Rabbit | 1:50 | Chemicon |
| Brachyury | Goat | 1:100 | SCBT |
| Sox17 | Rabbit | 1:100 | SCBT |
| β-tubulin | Rabbit | 1:1000 | Babco |
| Map2 | Mouse | 1:50 | Chemicon |
| Troponin | Mouse | 1:100 | DSHB |
| MLC | Mouse | 1:100 | DSHB |
| Albumin | Goat | 1:250 | Gift |
| Cyp7a1 | Goat | 1:250 | Gift |
| GFP | Rabbit | 1:200 | Millipore |
| H3K4me3 | Rabbit | 1:100 | Abcam |
| H3K27me3 | Mouse | 1:50 | Abcam |
