# Supplementary material for: Nucleic Acid and Non-Nucleic Acid-Based Reprogramming of Adult Limbal Progenitors to Pluripotency
Source: PLoS One. 2012 Oct 8;7(10):e46734. doi: 10.1371/journal.pone.0046734 (PMC3466310; doi:10.1371/journal.pone.0046734)
Supplement: Table S3 — List of primers for Bisulfite Sequencing. (DOC) [file pone.0046734.s007.doc]

**Table S3: List of primers for Bisulfite Sequencing**

| **Promoter** | **Primer sequence** | **Annealing temperature** | **Product size** |
| --- | --- | --- | --- |
| Oct4 | GGTTTTTTAGAGGATGGTTGAGTG  TCCAACCCTACTAACCCATCACC | 64 | 473 |
| Nanog | GATTTTGTAGGTGGGATTAATTGTGAATT  ACCAAAAAAACCCACACTCATATCAATATA | 64 | 367 |
