# Supplementary material for: Nucleic Acid and Non-Nucleic Acid-Based Reprogramming of Adult Limbal Progenitors to Pluripotency
Source: PLoS One. 2012 Oct 8;7(10):e46734. doi: 10.1371/journal.pone.0046734 (PMC3466310; doi:10.1371/journal.pone.0046734)
Supplement: Table S4 — List of primers for Chromatin Immunoprecipitation. (DOC) [file pone.0046734.s008.doc]

**Table S4: List of primers for Chromatin Immunoprecipitation**

| **Promoter** | **Primer sequence** | **Annealing temperature** | **Product size** |
| --- | --- | --- | --- |
| Oct4 | ATCCGAGCAACTGGTTTGTG  CAATCCCACCCTCTAGCCTT | 58 | 241 |
| Nanog | TCTTTAGATCAGAGGATGCCCCCTAAGC AAGCCTCCTACCCTACCCACCCCCTAT | 60 | 189 |
